# Measured spectrum environment map dataset with multi-radiation sources in urban scenarios

**DOI:** 10.1016/j.dib.2025.111909

**Published:** 2025-07-20

**Authors:** Haotian Zou, Qiuming Zhu, Qianhao Gao, Jie Wang, Zhipeng Lin, Yang Huang, Qihui Wu, Weizhi Zhong

**Affiliations:** a The College of Electronic and Information Engineering, Nanjing University of Aeronautics and Astronautics, Nanjing, PR China; b The College of Astronautics, Nanjing University of Aeronautics and Astronautics, Nanjing, PR China

**Keywords:** Spectrum environment map, Radio map, Spectrum strength, Radiation source, Propagation channel

## Abstract

This paper presents a measured spectrum strength dataset in the urban scenario with multiple radiation sources, aiming to address the limitation of open datasets for spectrum environment map (SEM) in realistic multi-source dynamic scenarios. The dataset was collected through high-precision measurements, covering the 30 MHz, 115 MHz, and 2 GHz frequency bands, with a spatial resolution of 1m×1 m. It includes spectrum strength or received signal strength (RSS) data in dBm for 80×105 grids. Each grid includes the information such as longitude, latitude, altitude, and time. The experiment utilizes three radiation sources with isotropic antennas and a mobile signal receiving system equipped with a spectrum analyzer and a GPS module. It collects data along a pre-defined path at a constant speed. The key feature of this dataset is its realistic representation of nonline ar characteristics of propagation channel in a multi-radiation source coexistence scenario. Its applications include the verification of spectrum map completion algorithms, wireless channel modelling, deep learning-driven signal prediction, and the optimization of Wi-Fi/cellular networks.

Specifications Table

**Table:** 

Subject	Computer Sciences
Specific subject area	Telecommunication Engineering
Type of data	Raw dataset, Table
Data collection	The hardware used in this article includes three transmission systems with isotropic antennas and one mobile signal receiving system. Each set of transmission system consists of one power supply, one signal generator and one isotropic antenna. The signal receiving system contains one spectrum analyzer, one laptop equipped with 32GB memory and an Intel Core i5–12500H processor, one isotropic antenna and one GPS module. The software used is independently developed using the graphical user interface (GUI) development tool of MATLAB. Multiple signal measurement experiments were conducted in urban scenarios with multiple radiation sources. The collected data are stored in .csv and .mat file formats.
Data source location	Institution: Nanjing University of Aeronautics and Astronautics City/Town/Region: Jiangning District, Nanjing City Country: China Latitude and longitude (GPS coordinates) for collected samples/data: 118.795806, 31.943062
Data accessibility	Repository name: Mendeley Data Data identification number: 10.17632/9wfy8pcxdb.1 Direct URL to data: https://data.mendeley.com/datasets/9wfy8pcxdb/1
Related research article	Compared to [12], which uses a dataset from a similar scenario at a single frequency (1300 MHz) and lacks detailed measurement procedures, this work provides more comprehensive multi-frequency measurements, along with thorough documentation of the measurement setup and parameters.

## 1 Value of the Data

• This dataset provides the measured spectrum strength data of urban scenarios, covering the RSS distribution at different spatial positions. It provides reliable empirical support for the study of propagation channel characteristics. [[1], [2]]
• The SEM information provided by this dataset is helpful for conducting comparative analysis and evaluation of existing and new algorithms for SEM completion and prediction. [[3], [4], [5]]
• This dataset is collected based on consistent hardware and software setup, with file formats of .csv and .mat. This ensures the reliability and reproducibility of the data, enabling it to provide high-quality measured data for both the academic community and the industrial sectors.
• The dataset can facilitate the optimization of wireless networks in intelligent urban environments, such as the deployment of Wi-Fi, cellular networks and sensor networks. Through RSS analysis, it can enhance the coverage effect of wireless networks and improve user experience and system performance. Moreover, by integrating with AI models for smart cities, this dataset can support intelligent tasks such as interference detection and dynamic spectrum allocation, thereby enabling more efficient wireless network management in smart cities. [[6], [7]]
• This dataset reflects the real-world spectrum distribution under scenarios of multi-source coexistence, providing a valuable reference for regulatory planning for electromagnetic interference and the formulation of spectrum policies for next-generation communication systems.

## 2 Background

With the rapid advancement of wireless communication technologies, the Internet of Things (IoT) and low-altitude aviation operations, the efficient management of electromagnetic spectrum resources has become increasingly challenging [[8], [9]]. The spectrum environment map (SEM), serving as an intuitive depiction of spatial electromagnetic field distribution, has proven to be of significant value in domains including dynamic spectrum sharing, interference mitigation, and radio environment perception [10]. This is particularly relevant given the pressing application requirements in unmanned aerial vehicle (UAV) communication, IoT node localization, and 5G/6 G network optimization[11]. However, existing publicly available datasets predominantly focus on signal acquisition from single radiation sources or static environments. In contrast, real-world complex electromagnetic environments commonly feature multiple radiation sources (e.g., base stations, Wi-Fi access points, sensor nodes). Furthermore, simulation-based data derived from theoretical propagation models often falls short in accurately capturing the channel characteristics in complex environments. Consequently, the development of a high-precision multi-radiation source RSS measured dataset can provide support for channel propagation characteristics, SEM construction and prediction algorithms, and urban wireless network optimization, etc.

## 3 Data Description

**Fig. 1:**
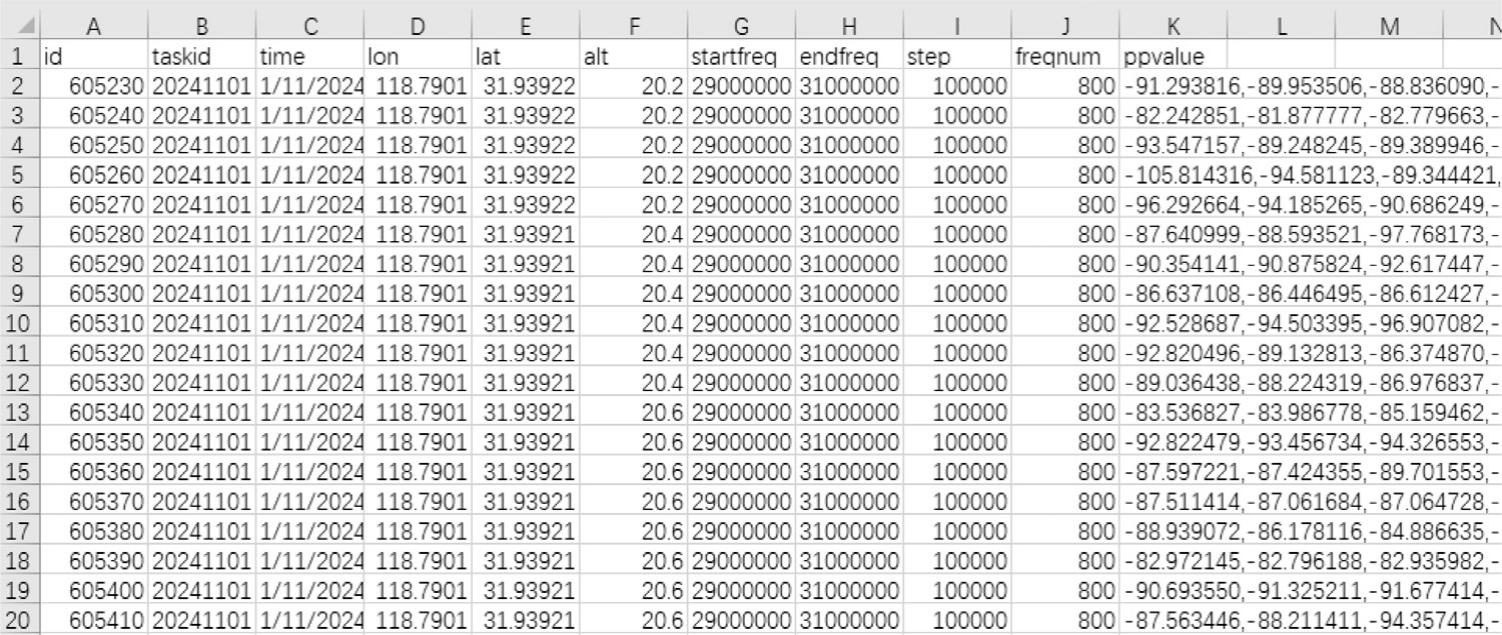
Snapshot of the .csv file from the 30 MHz experiment.

This research, targeting the complex electromagnetic environment with multiple radiation sources, proposes a measured RSS dataset for SEM construction. RSS data of 30 MHz, 115 MHz and 2 GHz were collected using measurement equipment and stored in real time. The stored data were saved in CSV format and included longitude, latitude, altitude, time, collected frequency points, and RSS. Detailed data specifications are provided in Table 1 and the data format is shown in Fig. 1.

**Table 1:** Description of each field of the collected data.

Field	Description
id	The number of each piece of data collected
taskid	Id of the collection task
time	The exact time when the data was received.
lon	The longitude of the receiver at the time of receiving this data
lat	The latitude of the receiver at the time of receiving this data
alt	The altitude of the receiver at the time of receiving this data
startfreq	The starting frequency of data collection
endfreq	The termination frequency of the data collection
step	The frequency interval of data acquisition within the set frequency band
freqnum	The number of frequency points collected in the set frequency band
ppvalue	The collected received signal strength data

**Fig. 2:**
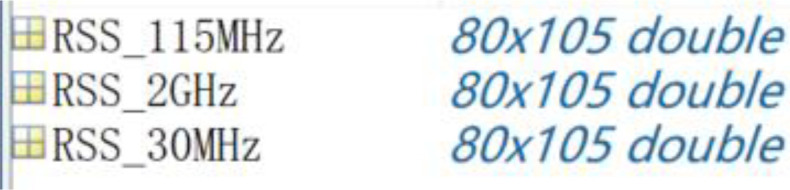
Snapshot of MATLAB workspace.

The SEM of the urban scenario is stored in .mat format, which is obtained by processing the original .csv files. The RSS data collected is stored in a 80×105 matrix, where x and y represent the spatial grid dimensions, and the values are of double type, indicating the signal strength (unit: dBm). Each file is about 18 KB in size. These files are suitable for the data call requirements of scientific computing environments such as MATLAB. Users can directly access the data content through the import function of the corresponding platform. Fig. 2 shows a screenshot of the MATLAB workspace after the file is imported.

This dataset is published under the CC BY 4.0 license, allowing free sharing and use provided the original authors are credited. The dataset complies with the GB/T 19710.1-2023 and GB/T 31074-2014 metadata standards.

## 4 Experimental Design, Materials and Methods

### 4.1 Hardware setup

**Fig. 3:**
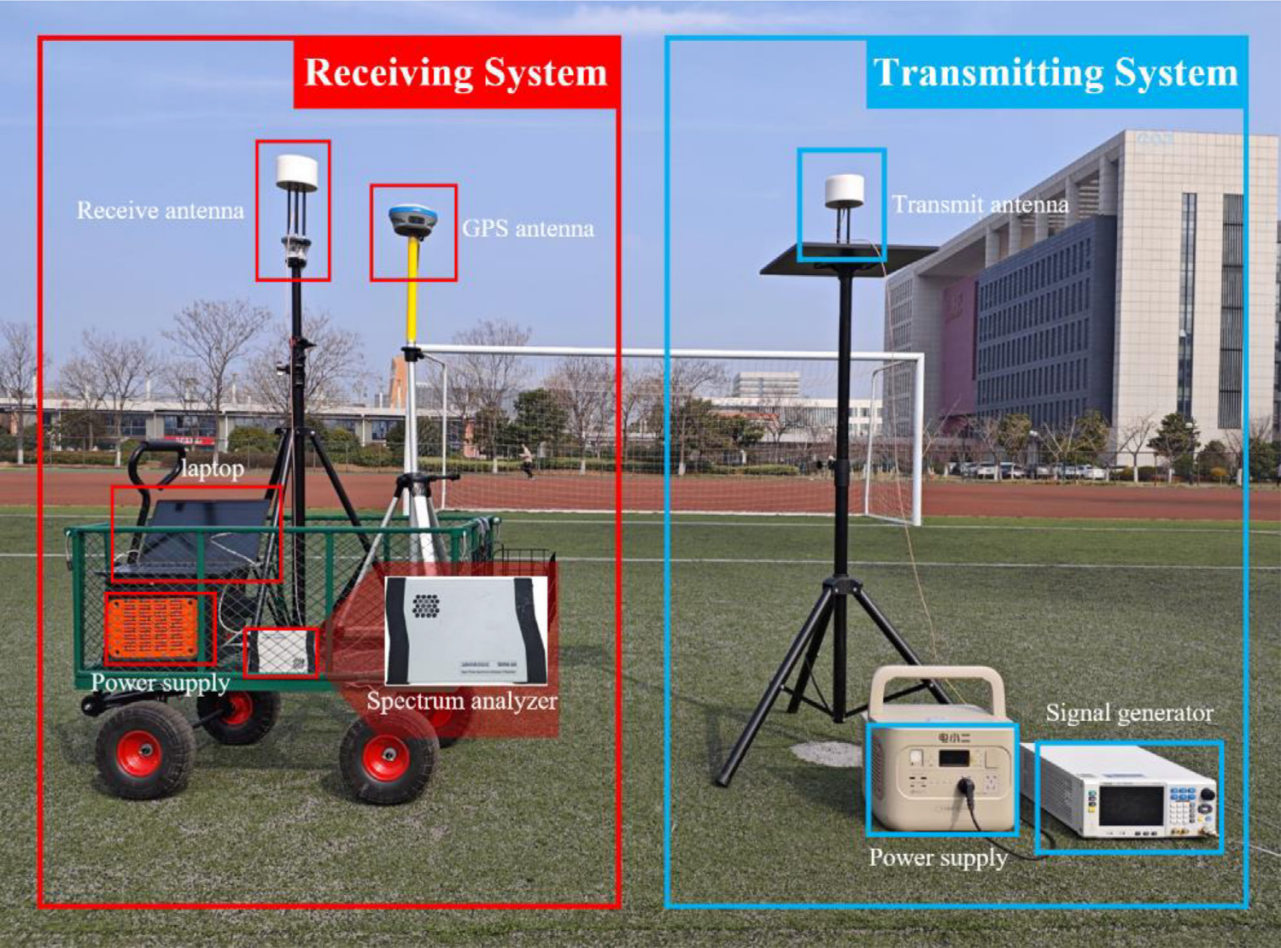
Hardware setup for data collection.

The measurement device consists of three sets of signal transmission systems and one set of signal receiving system. Each set of signal transmission system includes a power supply, a signal generator and an isotropic antenna, as shown in Fig. 3. The signal receiving system comprises: a spectrum analyzer, a laptop with 32GB memory and an Intel Core i5–12500H processor, an isotropic antenna and a GPS module.

### 4.2 Software setup

The software is independently developed using the graphical user interface (GUI) development tool of MATLAB. This software aims to achieve efficient processing, situation reconstruction, and performance evaluation of collected spectrum data. The software consists of two parts: the data acquisition module and the SEM construction module.

In the data acquisition module, parameters such as the starting frequency, ending frequency, and frequency interval of the collected signals can be set to determine the spectrum range and resolution. The detailed configuration parameters of the spectrum analyzer are presented in Table 2.

**Table 2:** Configuration parameters of the spectrum analyzer.

Parameter category	Configuration value
Center frequency	30 MHz\115 MHz\2.0 GHz
Starting frequency	29 MHz\114 MHz\1.999 GHz
Termination frequency	31 MHz\116 MHz\2.001 GHz
Resolution bandwidth	100 kHz
Video bandwidth	10 kHz
Reference level	0 dBm
Detector	RMS

Additionally, to compensate for the energy loss of the signals during their transmission within the equipment (such as transmission lines, amplifiers, and connectors.), the collected data is corrected. According to the signal propagation model, the received signal power can be expressed as

(1) $$P_{r}\left(\text{dBm}\right)=P_{t}\left(\text{dBm}\right)+G_{t}\left(\text{dB}\right)+G_{r}\left(\text{dB}\right)-PL\left(\text{dB}\right)-X\left(\text{dB}\right)$$

where $P_{t}$ is the transmitted signal power, $G_{t}$ and $G_{r}$ are the gains of the transmitting and receiving antennas, PL is the path loss, and X is the system loss that needs to be obtained. Based on the free-space propagation model, the path loss PL can be calculated as

(2) PL=32.44+20log(f)+20log(d)

where f is the transmission frequency, and d is the distance between the transmitter and the receiver. Accordingly, the system loss X can be calculated to adjust the measured RSS values, ensuring that the corrected data more accurately reflects the real signal strength.

The SEM construction module can construct a complete spectrum environment map using the completion algorithms based on the collected RSS data.

### 4.3 Experimental procedure

**Fig. 4:**
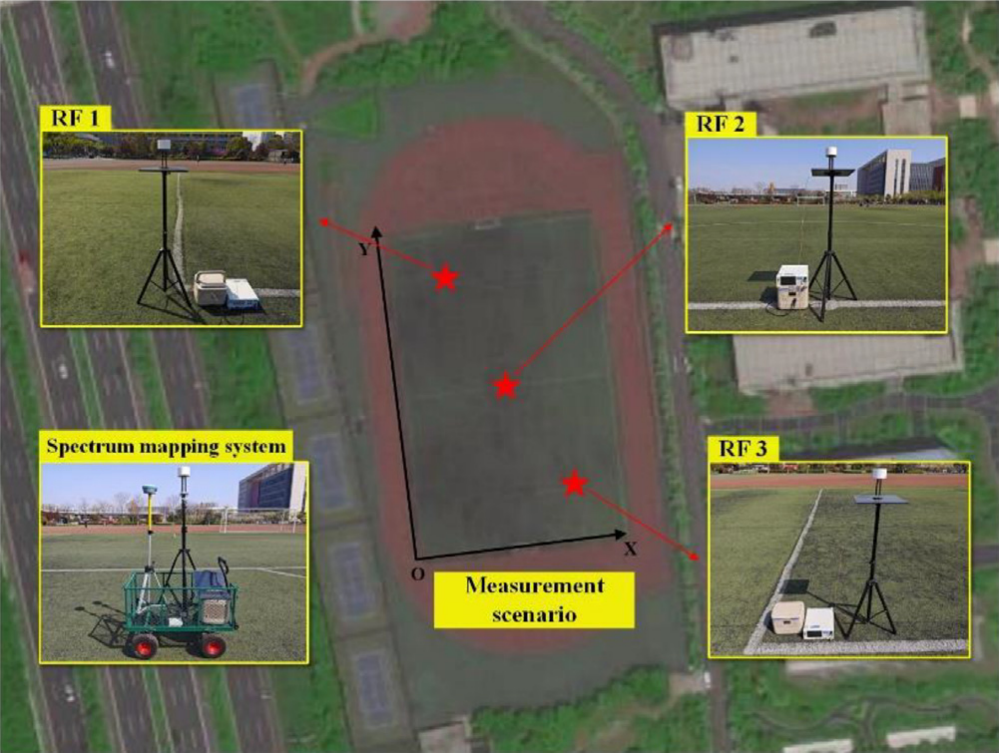
Different realistic views of the playground for conducting experiments.

The measurement area covers a rectangular region of 80 *m* × 105 m, consisting of different types of buildings with heights from 10–30 m, open grassland, trees, and roads. The tests were conducted in the morning under favorable weather conditions. As illustrated in Fig. 4, three sets of signal transmission systems are sequentially deployed at the designated positions in the measurement area. Table 3 summarizes all the important parameters. The RSS is collected in real time by a signal receiving system. It moves at a constant speed of 1 m per second along an ``S'' shaped route, starting from one end of measurement area until the entire area is covered. The signal receiving system traverses the measurement area exhaustively without path repetition, ensuring comprehensive yet non-redundant data collection for precise signal strength mapping.

**Table 3:** Measurement parameters.

	parameter values					
	Serial Number	height (m)	Horizontal position (m)	Vertical position (m)	Power (dBm)	Antenna type
Source configuration	1	0.6	28	90	10	isotropic
	2	0.6	50	58	10	isotropic
	3	0.6	78	25	10	isotropic
Measured region	80m*105m					
Signal frequency	2GHz,115MHz,30MHz					
Receiving system’s height	1m					
Grid size	1m*1m					

**Fig. 5:**
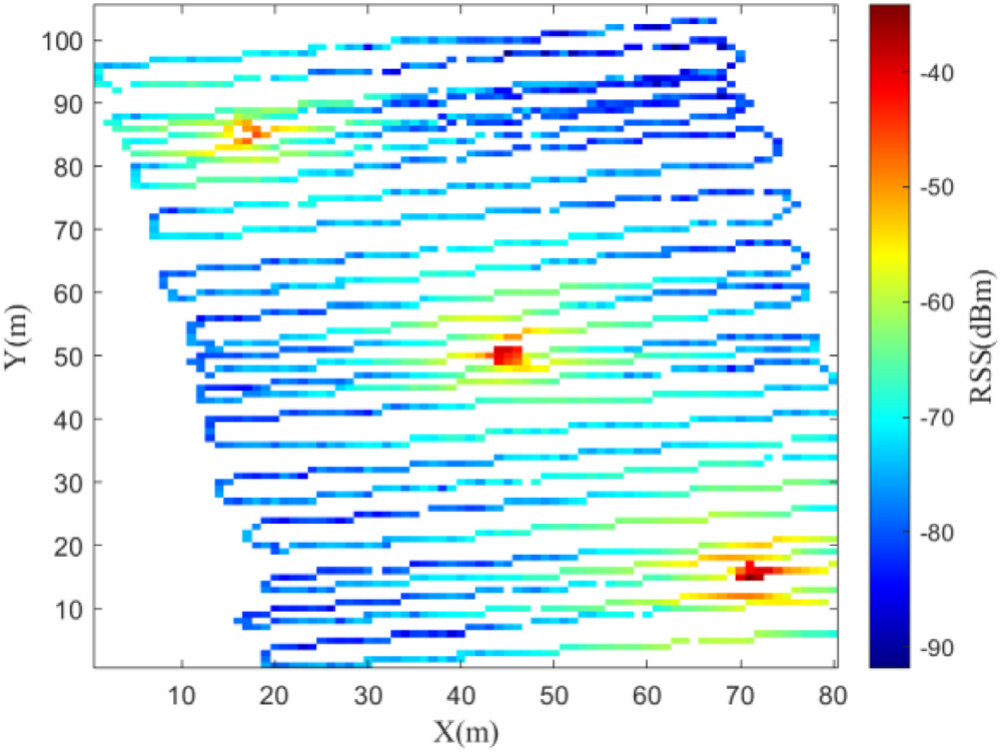
Trajectory of data collection in the 30 MHz experiment.

**Fig. 6:**
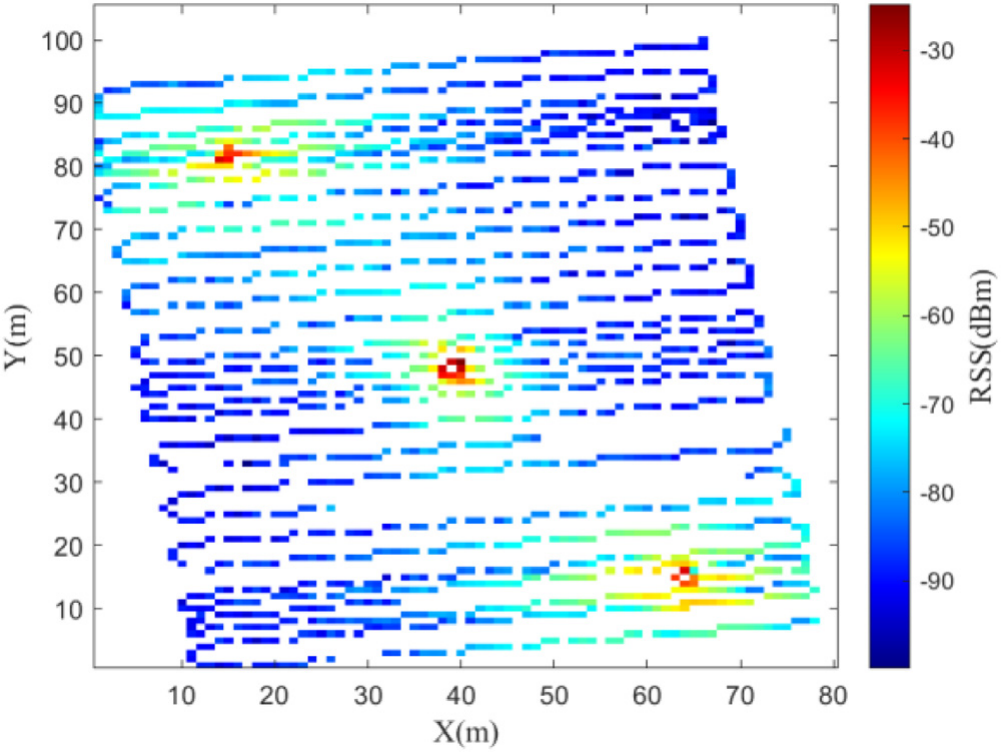
Trajectory of data collection in the 115 MHz experiment.

**Fig. 7:**
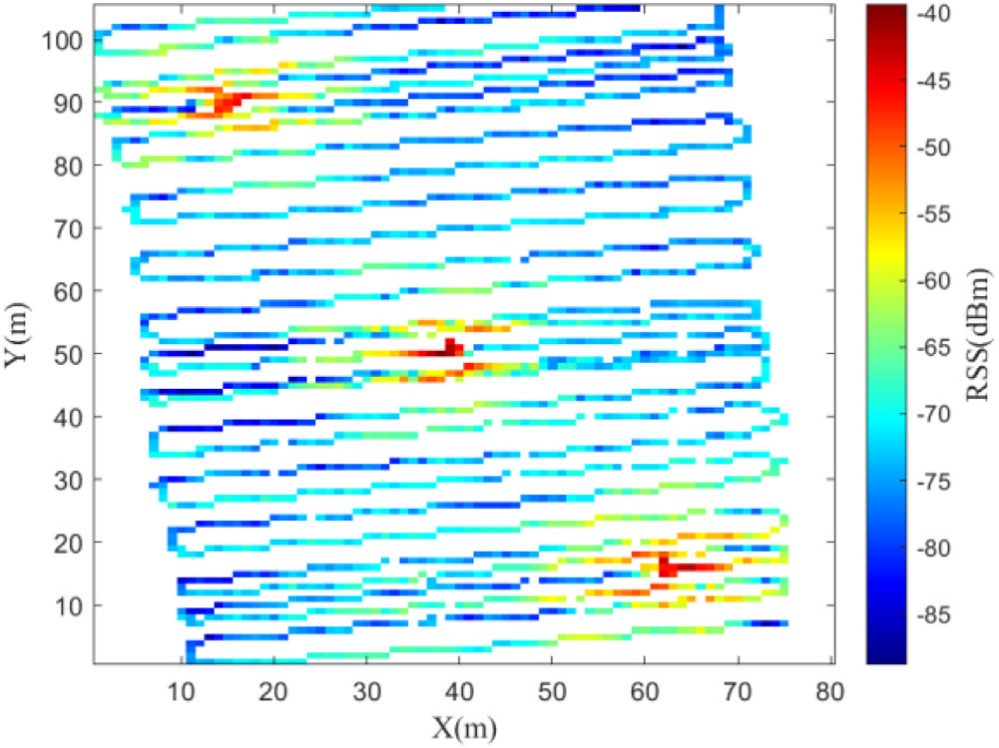
Trajectory of data collection in the 2 GHz experiment.

In experiments with different frequencies, the positions remain fixed. The frequency of signal generator and spectrum analyzer are changed. Fig. 5, Fig. 6, Fig. 7 show the trajectories in the field when the frequencies are set to 30 MHz, 115 MHz, and 2 GHz respectively. The entire map is regarded as 80×105 grids, with each grid cell colored according to its RSS value. Each grid represents a size of 1 m*1 m in reality.

This dataset can be used for the construction and prediction of SEMs, providing data training, performance verification and evaluation benchmarks for different algorithms. Additionally, it can be utilized to study wireless channel modeling, propagation characteristic analysis and communication environment cognition, as well as network optimization and resource management.

**Fig. 8:**
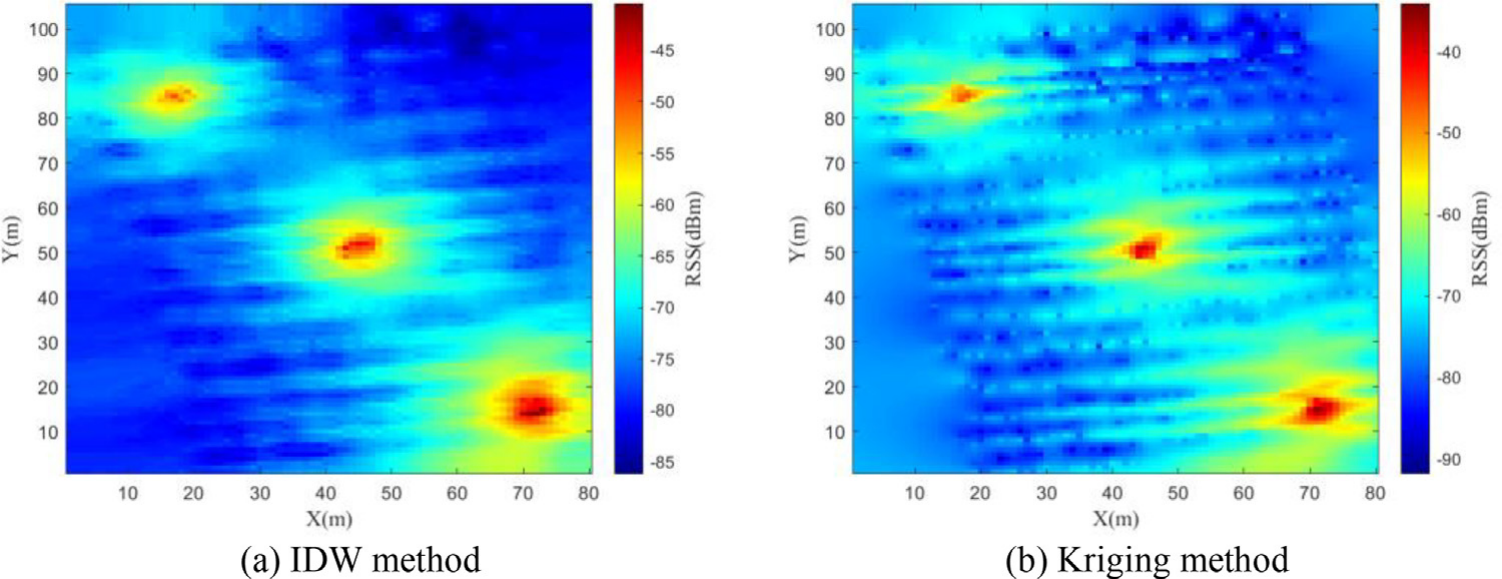
Constructed REM at 30MHz.

**Fig. 9:**
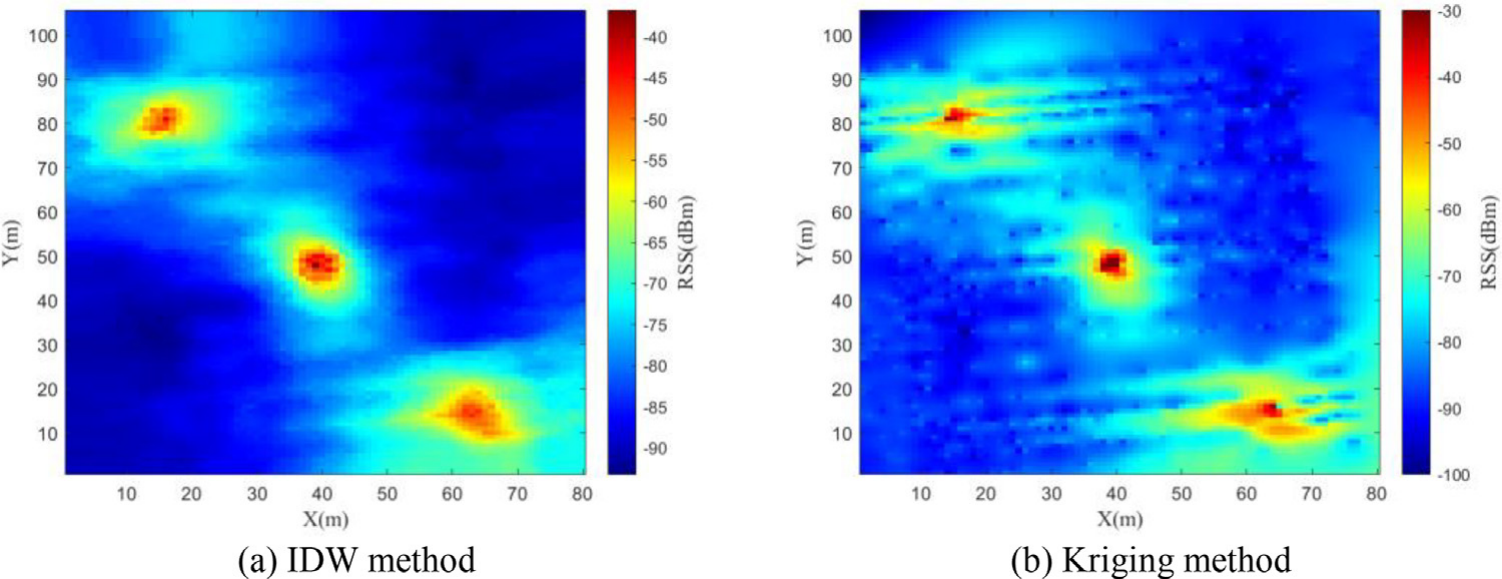
Constructed REM at 115MHz.

**Fig. 10:**
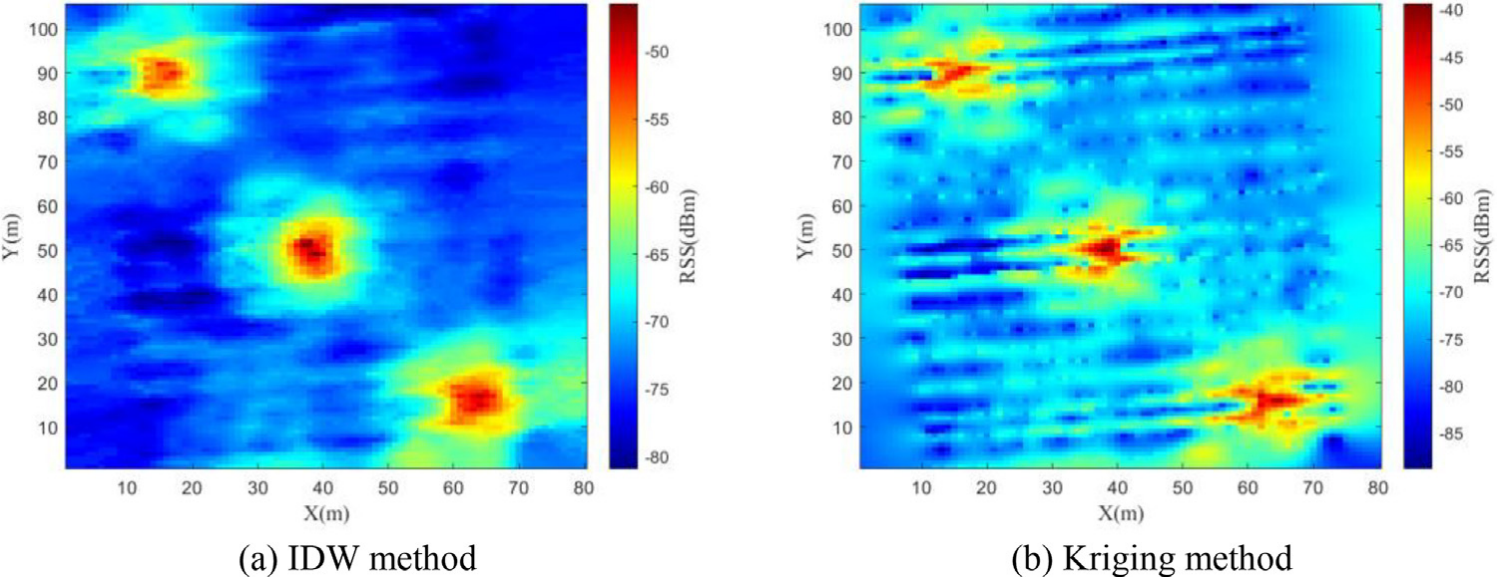
Constructed REM at 2GHz.

In the research of constructing SEMs based on this dataset, we adopted the inverse distance weighting (IDW) and kriging interpolation methods within the SEM construction module. Fig. 8, Fig. 9, Fig. 10 respectively present the constructed SEMs of 30 MHz, 115 MHz and 2 GHz frequency bands. The experimental results demonstrate that the SEM constructed using IDW method significantly outperforms the Kriging interpolation method in terms of reconstruction accuracy and computational efficiency.

## Limitations

None.

## Ethics Statement

The authors have read and follow the ethical requirements for publication in Data in Brief and confirming that the current work does not involve human subjects, animal experiments, or any data collected from social media platforms.

## CRediT Author Statement

**Haotian Zou:** Data curation, Writing - original draft, Software. **Qiuming Zhu:** Conceptualization, Methodology, Writing - review & editing. **Qianhao Gao:** Software, Validation. **Jie Wang:** Data curation, Investigation. **Zhipeng Lin:** Resources. **Yang Huang:** Resources. **Qihui Wu:** Supervision. **Weizhi Zhong:** Project administration.

## Data Availability

Mendeley DataMeasured Radio Map Dataset with Multi-radiation Sources of Urban Scenarios (80m×105m) (Original data).
